# Genetic variants of nuclear factor erythroid‐derived 2‐like 2 associated with the complications in Han descents with type 2 diabetes mellitus of Northeast China

**DOI:** 10.1111/jcmm.12900

**Published:** 2016-07-04

**Authors:** Xiaohong Xu, Jing Sun, Xiaomin Chang, Ji Wang, Manyu Luo, Kupper A. Wintergerst, Lining Miao, Lu Cai

**Affiliations:** ^1^ Department of Gynecology and ObstetricsSecond Hospital of Jilin UniversityChangchunChina; ^2^ Department of NephropathySecond Hospital of Jilin UniversityChangchunChina; ^3^ Kosair Children's Hospital Research InstituteDepartment of PediatricsUniversity of LouisvilleLouisvilleKYUSA; ^4^ Division of EndocrinologyDepartment of PediatricsUniversity of LouisvilleLouisvilleKYUSA; ^5^ Wendy L. Novak Diabetes Care CenterKosair Children's HospitalUniversity of LouisvilleLouisvilleKYUSA

**Keywords:** NFE2L2 gene mutation, diabetes, diabetic complications, Chinese population, Nrf2 polymorphism

## Abstract

The transcription factor nuclear factor erythroid 2‐like 2 (NFE2L2) is essential for preventing type 2 diabetes mellitus (T2DM)‐induced complications in animal models. This case and control study assessed genetic variants of NFE2L2 for associations with T2DM and its complications in Han Chinese volunteers. T2DM patients with (*n* = 214) or without (*n* = 236) complications, or healthy controls (*n* = 359), were genotyped for six NFE2L2 single nucleotide polymorphisms (SNPs: rs2364723, rs13001694, rs10497511, rs1806649, rs1962142 and rs6726395) with TaqMan Pre‐Designed SNP Genotyping and Sequence System. Serum levels of heme oxygenase‐1 (HMOX1) were determined through enzyme‐linked immunosorbent assay. Informative data were obtained for 341 cases and 266 controls. Between T2DM patients and controls, the genotypic and allelic frequencies and haplotypes of the SNPs were similar. However, there was a significant difference in genotypic and allelic frequencies of rs2364723, rs10497511, rs1962142 and rs6726395 between T2DM patients with and without complications, including peripheral neuropathy, nephropathy, retinopathy, foot ulcers and microangiopathy. Furthermore, HMOX1 levels were significantly higher in T2DM patients with complications than in controls. Multiple logistic regression analysis, however, showed that only rs2364723 significantly reduced levels of serum HMOX1 in T2DM patients for the GG genotype carriers compared with participants with CG+CC genotype. The data suggest that although NFE2L2 rs2364723, rs10497511, rs1962142 and rs6726395 were not associated with T2DM risk, they were significantly associated with complications of T2DM. In addition, only for rs2364723 higher serum HMOX1 levels were found in the T2DM patients with CG+CC than those with GG genotype.

## Introduction

Type 2 diabetes mellitus (T2DM) is characterized by insulin resistance, with or without defects in insulin production and secretion. Globally, up to 90% of all diabetes cases are T2DM, and the disease is a leading world heath challenge 1, 2. In China, the prevalence of diabetes is rising at an alarming rate. In the year 2010, 11.6% of a representative sample of Chinese adults had T2DM, translating to as many as 113.9 million in the nation 1. It was estimated that 382 million individuals suffered from diabetes in 2013, a number that could rise to 592 million by 2035 2. These findings indicate the importance of diabetes as a public health problem in the world and in China.

To date, there is no effective treatment for T2DM. Clinically, T2DM management consists of changes in lifestyle, lowering other cardiovascular risk factors and maintaining blood glucose levels in the normal range 3. Routine medical treatment is challenging, especially the identification and management of complications associated with micro‐ and macrovascular damage in diabetes.

The development of T2DM and its complications are likely because of multiple factors, including the lifestyle and genetic changes 4, 5, 6. The most common type of genetic variations is single nucleotide polymorphisms (SNPs), which have been shown to impact population susceptibility to diseases, and individual response to drug treatments. Many SNPs are silent (often called synonymous SNPs), that is, they have no direct effect on protein coding. However, some silent SNPs can be used as genetic markers of adjacent functional variants that contribute to disease by virtue of linkage disequilibrium. Other SNPs that affect the coding or regulatory regions of genes (usually promoter regions) may have direct functional consequences 4, 5, 7.

To date, there have been numerous studies regarding links between SNPs and the risk of T2DM. Through genetic association research, some SNPs of different genes have been found to increase the risk of developing T2DM in certain populations 4, 5. In terms of the mechanism by which diabetes is induced and diabetic complications were developed, there are a few possibilities that have been proposed; however, a generally appreciated one is the oxidative stress caused by either overgeneration of reactive oxygen species or reduction in anti‐oxidative mechanisms 3, 6, 8, 9.

The protein nuclear factor erythroid 2‐like 2 (NFE2L2, also known as Nrf2; encoded by the NFE2L2 gene) is a basic leucine zipper 10 to play a master regulatory role in redox balance in the cytoprotective response 10, 11. Many experimental studies have suggested that NFE2L2 plays a preventive role in the development of both T1DM and T2DM 12 as well as their complications such as diabetic cardiomyopathy, nephropathy 6, 13, neuropathy and retinopathy 14, 15. However, it remains unknown whether NFE2L2 polymorphisms are associated with the risk of diabetes and diabetic complications.

During non‐stressed conditions, NFE2L2 is sequestered in the cytoplasm by the repressor protein KEAP1 (kelch‐like ECH [erythroid cell‐derived protein with CNC homology]‐associated protein 1) and targeted for proteasomal degradation 10, 11. However, during oxidative stress NFE2L2 is translocated from the cytoplasm into the nucleus, where it activates transcription of a large battery of genes by binding to antioxidant response elements (AREs) in the upstream promoter regions of its downstream genes 6, 10, 11. The activation of NFE2L2 leads to production of cytoprotective proteins including NAD[P]H dehydrogenase, quinone 1 (NQO1), glutamate‐cysteine ligase and heme oxygenase‐1 (HMOX1) 10, 16. The antioxidant response provided by the NFE2L2 and KEAP1‐NFE2L2/ARE signalling pathways protect the pulmonary, hepatic, digestive, neural and cardiovascular systems 10, 11, 16.

In this study, therefore, we investigated NFE2L2 polymorphisms for associations with risk of T2DM and its complications in a cohort of Chinese volunteers of Han descent. We also investigated whether NFE2L2 SNPs are related to expression of its downstream gene, HMOX1.

## Materials and methods

### Study population

For this study we recruited volunteers of Han Chinese descent: 450 patients (214 cases with complications and 236 cases without complications) and 359 healthy controls with the same age range (Table 1). The participants underwent genotyping analysis performed at the Research Center for Genomic Medicine of Jilin University (Changchun, China) between January 2010 and January 2012. A diagnosis of T2DM was determined in accordance with the diagnostic criteria set by the American Diabetes Association (ADA) in 1999 17. These patients were also checked with the updated ADA diagnostic criteria of T2DM and they are mostly consistent with the latest standards 18. For the study participants, the inclusion criteria were as follows: no history of receiving pharmacologic treatment for diabetes, no clinically systemic diseases and no other acute or chronic inflammatory diseases, cancer and or acute respiratory infection when T2DM was diagnosed. This study was approved by the Second Hospital Ethics Committee of Jilin University [Protocol #: 2013L01477, (2014)临会审第(018)号] and written informed consent was obtained from all individuals. Diagnosed complications (after clinical examinations) in the group of T2DM patients with complications were developed after they were diagnosed diabetes, and included diabetic foot ulcer, nephropathy, retinopathy, microangiopathy, peripheral neuropathy or neurogenic bladder.

**Table 1:** Demographics and clinical characteristics of T2DM patients and healthy control participants

	Control	T2DM	*P*‐value
Diagnosis, *n* (%)	359 (44.4)	450 (55.6)	0.876
Age, years	55.7 ± 10.7	55.9 ± 13.2	0.746
Gender, male/female	220/139	250/200	0.115
BMI, kg/m^2^	25.6 ± 2.7	25.7 ± 3.2	0.724
Total cholesterol, mmol/l	4.37 ± 1.16	4.62 ± 1.23	0.003
Total triglyceride, mmol/l	1.73 ± 1.16	1.81 ± 0.61	0.033
High‐density lipoprotein, mmol/l	1.03 ± 0.23	1.02 ± 0.23	0.810
Low‐density lipoprotein, mmol/l	3.39 ± 0.39	3.44 ± 0.62	0.144
Diabetic foot, *n*	0	8	–
Diabetic nephropathy, *n*	0	85	–
Diabetic microangiopathy, *n*	0	9	–
Diabetic retinopathy, *n*	0	33	–
Diabetic peripheral neuropathy, *n*	0	115	–
Diabetic neurogenic bladder, *n*	0	1	–
Hyperlipidaemia, *n*	0	158	–
Non‐alcoholic fatty liver disease, *n*	0	53	–

The T2DM patients were originally from Northeast China, and the age‐matched healthy control participants (with neither T2DM nor family history of diabetes) were randomly selected from the health examination clinics of our hospital. During enrolment, each participant filled out a questionnaire regarding health, diagnosis of type of diabetes and T2DM duration, family history of diabetes and ethnic background (Table 1). Clinical exams included height and weight to determine body mass index (BMI; kg/m^2^).

### SNP selection and TaqMan SNP genotyping

We used the website of the International HapMap (Haplotype Mapping) Project (http://www.hapmap.org) to download NFE2L2 SNP data from the Han Chinese in Beijing database. Data were processed with Haploview software 4.2.0.0 (Cambridge, MA, USA). Linkage disequilibrium blocks were constructed in accordance with a previous study 19. The tag SNPs were assigned by the tagger function of Haploview. A minor allele frequency of ≥5% and pairwise tagging with a minimum *r* ^2^ of 0.80 were applied to capture the common variations within the blocks covering NFE2L2. Most of these sites were also checked in PubMed for its consistency with published studies 20, 21. We therefore obtained six NFE2L2 SNPs (Table 3), including rs2364723 (SNP loci, 178126546; genotype, G/C), rs13001694 (178118990; A/G), rs10497511 (178119296; G/A), rs1806649 (178118125; C/T), rs1962142 (178113484; G/A) and rs6726395 (178103229; G/A).

To genotype volunteers for these SNPs, 5 ml of peripheral blood was collected from each into heparin‐containing tubes. These blood samples were immediately stored at −20°C until further processed. Genomic DNA was extracted from the samples with a Wizard Genomic DNA Purification Kit (Promega, Madison, WI, USA) in accordance with the manufacturer's protocol, and quantified by Nanodrop (Thermo‐Fisher, Waltham, MA, USA).

The Tag SNPs were genotyped with TaqMan Pre‐Designed SNP genotyping kits (Applied Biosystems, Foster City, CA, USA) and the ABI PRISM 7300 Sequence Detection System (Applied Biosystems). The polymerase chain reaction (PCR) amplification was performed in a 25 μl reaction mixture under the following conditions: 40 cycles of 95°C for 10 min., 92°C for 15 sec. and 60°C for 1 min. The primers to detect NFE2L2 SNPs were synthesized by Applied Biosystems. The ABI probe codes were C_351878_10for rs2364723; C_31613510_10 for rs13001694; C_30175556_20 for rs10497511; C_11634983_10 for rs1806649; C_11634984_10 for rs1962142 and C_155538_10 for rs6726395.

The PCR products were randomly selected for DNA sequencing analysis by Shanghai Sangon Biological Engineering Technology & Services. For each SNP we choose three cases and the results were compared with the results of Taqman genotyping.

### Measurement of serum HMOX1 levels

Serum HMOX1 levels were measured in 111 randomly selected T2DM patients (27 with complications and 84 without complications) and 53 controls with commercial ELISA kits (Enzo Life Sciences, Exeter, UK) in accordance with the manufacturer's protocol.

### Statistical analysis

The genotype frequency of NFE2L2 was assessed for Hardy–Weinberg equilibrium by the chi‐squared goodness‐of‐fit test. A *P*‐value >0.05 indicated Hardy–Weinberg equilibrium. Genotype frequency and distribution in patients and controls were analysed by the chi‐squared test with statistical software Statistical Packages for Social Sciences (SPSS12.0, Chicago, IL, USA). The association between the combined effects of NFE2L2 SNPs and T2DM (with or without diabetic complications) was analysed by SHEsis software as in a previous study 22.

Differences in serum HMOX1 levels or SNPs between patients and controls were analysed by Student's *t*‐test and analysis of variance, respectively, and the data presented as mean ± S.D. Multivariate logistic regression analysis for the influence of clinical and genetic factors for serum HMOX1 levels was also performed. A *P*‐value ≤0.05 was considered statistically significant.

## Results

### Characteristics of the studied volunteers

The mean ages of the T2DM (55.9 ± 13.2 years) and control participants (55.7 ± 10.3 years) were similar, as were the gender distributions and BMI (25.7 ± 3.2 and 25.6 ± 2.7 kg/m^2^, respectively). The total cholesterol and total triglyceride in T2DM (4.62 ± 1.23 mmol/l and 1.81 ± 0.61 mmol/l, respectively) were higher than control participants(4.37 ± 1.16 mmol/l and 1.73 ± 1.16 mmol/l, *P* < 0.05, Table 1). There was no difference between T2DM patients and controls for high‐density lipoprotein and low‐density lipoprotein (Table 1).

Among 450 T2DM patients, 214 T2DM have various complications that were associated with diabetes, including peripheral neuropathy (*n* = 115), nephropathy (*n* = 85), retinopathy (*n* = 33), foot ulcers (*n* = 8), microangiopathy (*n* = 9) and neurogenic bladder (*n* = 1). There was no difference for age, gender, BMI, total cholesterol and high‐density lipoprotein (Table 2). However, total triglyceride (1.93 ± 0.60 mmol/l) and low‐density lipoprotein (3.60 ± 0.40 mmol/l) in the group without complications were significantly higher and low, respectively, than that of patients with complications (1.68 ± 0.60 mmol/l and 3.29 ± 0.73 mmol/l, *P* < 0.05, Table 2).

**Table 2:** Demographics and clinical characteristics of T2DM patients with and without complications

	T2DM with complications	T2DM without complications	*P*‐value
Diagnosis, *n* (%)	214 (47.6)	236 (52.4)	–
Age, years	56.9 ± 11.6	55.1 ± 14.5	0.144
Gender, male/female	121/93	129/107	0.688
BMI, kg/m^2^	25.7 ± 3.3	25.7 ± 3.2	0.803
Total cholesterol, mmol/l	4.57 ± 1.43	4.68 ± 1.09	0.356
Total triglyceride, mmol/l	1.68 ± 0.60	1.93 ± 0.60	0.000
High‐density lipoprotein, mmol/l	1.03 ± 0.23	1.03 ± 0.22	0.835
Low‐density lipoprotein, mmol/l	3.60 ± 0.40	3.29 ± 0.73	0.000
Diabetic foot, *n*	8	0	–
Diabetic nephropathy, *n*	85	0	–
Diabetic microangiopathy, *n*	9	0	–
Diabetic retinopathy, *n*	33	0	–
Diabetic peripheral neuropathy, *n*	115	0	–
Diabetic neurogenic bladder, *n*	1	0	–
Hyperlipidaemia	78	80	0.737
Non‐alcoholic fatty liver disease	28	25	0.495

### Genotyping distribution of NFE2L2 SNPs

The six NFE2L2 SNPs genotyped in this study were highly polymorphic and their genotypic distributions were in Hardy–Weinberg equilibrium (*P* > 0.05), suggesting the suitability of this sample pool for genetic analysis.

After genotyping the patients and control participants, we only analysed the data for 341 patients and 266 control, respectively (Table 3); for the remaining cases, the serum samples or PCR readings were abandoned if one of the six sites in the sample was failed to be genotyped. Our data showed no significant difference between the T2DM patients and controls with regard to genotypic or allelic frequencies of the six NFE2L2 SNPs (*P* > 0.05). However, there were significant differences both in genotypic frequencies and in allelic frequency of rs2364723, rs10497511, rs1962142 and rs6726395 between the T2DM patients without and with complications (*P* = 0.000–0.046, Table 4). There were also significant differences for both Dominants and Recessives of rs10497511, rs1962142 and rs6726395 between the patients with and without complications too (*P* = 0.001–0.019, Table 4).

**Table 3:** Genotype and allele frequency of the NFE2L2 SNPs between T2DM patients (*n* = 341) and controls (*n* = 266)

	T2DM, *n* (%)	Control, *n* (%)	OR (95% CI)	*P*
rs2364723				
GG	99 (0.29)	73 (0.27)	1^a^	–
GC	174 (0.51)	135 (0.50)	1.16 (0.73–1.84)	0.537
CC	68 (0.20)	58 (0.22)	1.10 (0.73–1.67)	0.656
HWE *P*‐value	0.592	0.766		
G	372 (0.54)	281 (0.53)	1^a^	–
C	310 (0.46)	251 (0.47)	1.07 (0.85~1.35)	0.55
rs13001694				
AA	259 (0.76)	200 (0.75)	1^a^	–
AG	77 (0.23)	64 (0.24)	1.93 (0.37–10.05)	0.435
GG	5 (0.015)	2 (0.08)	0.93 (0.64–1.36)	0.704
HWE *P*‐value	0.789	0.197		
A	595 (0.87)	464 (0.87)	1^a^	–
G	87 (0.13)	68 (0.13)	1.00 (0.71–1.41)	0.990
rs10497511				
AA	172 (0.50)	141 (0.53)	1^a^	–
AG	142 (0.42)	105 (0.40)	0.90 (0.49–1.68)	0.748
GG	27 (0.08)	20 (0.07)	1.00 (0.53–1.88)	0.996
HWE *P*‐value	0.758	0.941		
A	486 (0.71)	387 (0.73)	1^a^	–
G	196 (0.29)	145 (0.27)	1.03 (0.80–1.33)	0.824
rs1806649				
CC	279 (0.82)	213 (0.80)	1^a^	–
CT	59 (0.17)	52 (0.19)	0.44 (0.05–4.23)	0.474
TT	3 (0.009)	1 (0.004)	0.38 (0.04–3.75)	0.406
HWE *P*‐value	0.951	0.242		
C	617 (0.90)	478 (0.90)	1^a^	–
T	65 (0.10)	54 (0.10)	1.07 (0.73–1.57)	0.719
rs1962142				
GG	185 (0.54)	146 (0.55)	1^a^	–
GA	134 (0.39)	101 (0.38)	1.09 (0.57–2.10)	0.786
AA	22 (0.07)	19 (0.07)	1.15 (0.59–2.23)	0.689
HWE *P*‐value	0.409	0.789		
G	504 (0.74)	393 (0.74)	1^a^	
A	178 (0.26)	139 (0.26)	1.10 (0.85–1.43)	0.476
rs6726395				
AA	67 (0.20)	47 (0.18)	1^a^	–
AG	175 (0.51)	134 (0.50)	1.22 (0.76–1.96)	0.402
GG	99 (0.29)	85 (0.32)	1.12 (0.78–1.62)	0.541
HWE *P*‐value	0.512	0.642		
A	309 (0.45)	228 (0.43)	1^a^	–
G	373 (0.55)	304 (0.57)	1.07 (0.85–1.34)	0.580

a Reference category (odds ratio, 1.0); HWE: Hardy–Weinberg equilibrium.

**Table 4:** Genotype and allele frequencies of NFE2L2 SNPs stratified by T2DM complications

	T2DM ± complications		Co‐dominants (11 *versus* 12 *versus* 22)*		Dominants (11 *versus* 12 + 22)*		Recessives (22 *versus* 11 + 12)*	
	+, *n* (%)†	−, *n* (%)‡	OR (95% CI)	*P*	OR (95% CI)	*P*	OR (95% CI)	*P*
rs2364723 (G>C)								
GG	41 (0.25)	58 (0.33)	1§	–	0.67 (0.42–1.08)	0.100	1.57 (0.92–2.68)	0.100
GC	85 (0.52)	89 (0.51)	0.53 (0.28–0.98)	0.044¶				
CC	39 (0.24)	29 (0.16)	0.71 (0.40–1.25)	0.235				
G	167 (0.51)	205 (0.58)	1§	–				
C	163 (0.49)	147 (0.42)	0.74 (0.54–0.99)	0.046¶				
rs13001694 (G>A)								
AA	124 (0.75)	135 (0.77)	1§	–	0.26 (0.03–2.37)	0.233	0.92 (0.56–1.51)	0.737
AG	40 (0.24)	37 (0.21)	0.27 (0.03–2.47)	0.247				
GG	1 (0.006)	4 (0.023)	1.18 (0.71–1.96)	0.531				
A	288 (0.87)	307 (0.87)	1§	–				
G	42 (0.13)	45 (0.13)	1.01 (0.64–1.58)	0.982				
rs10497511 (A>G)								
AA	95 (0.58)	77 (0.44)	1§	–	1.75 (1.14–2.68)	0.011¶	2.89 (1.19–7.04)	0.019¶
AG	63 (0.38)	79 (0.45)	3.53 (1.42–8.77)	0.007¶				
GG	7 (0.04)	20 (0.11)	2.28 (0.91–5.73)	0.080				
A	253 (0.77)	233 (0.66)	1§	–				
G	77 (0.23)	119 (0.34)	1.68 (1.20–2.35)	0.003¶				
rs1806649 (C>T)								
CC	132 (0.80)	147 (0.84)	1§	–	1.27 (0.73–2.20)	0.400	0.53 (0.05–5.91)	0.605
CT	32 (0.19)	27 (0.15)	1.80 (0.16–20.03)	0.634				
TT	1 (0.006)	2 (0.01)	2.37 (0.20–27.59)	0.491				
C	296 (0.90)	321 (0.91)	1§	–				
T	34 (0.10)	31 (0.09)	1.19 (0.71–1.98)	0.506				
rs1962142 (G>A)								
GG	105 (0.64)	80 (0.46)	1§	–	0.48 (0.31–0.74)	0.001¶	0.22 (0.07–0.66)	0.007¶
GA	56 (0.34)	78 (0.44)	5.91 (1.92–18.13)	0.002¶				
AA	4 (0.02)	18 (0.10)	3.23 (1.04–10.07)	0.043¶				
G	266 (0.81)	238 (0.68)	1§	–				
A	64 (0.19)	114 (0.32)	1.26 (1.12–1.42)	0.000¶				
rs6726395 (A>G)								
AA	22 (0.13)	45 (0.26)	1§	–	2.23 (1.27–3.92)	0.005¶	1.79 (1.11–2.87)	0.017¶
AG	85 (0.52)	90 (0.51)	0.35 (0.18–0.66)	0.001¶				
GG	58 (0.35)	41 (0.23)	0.67 (0.41–1.10)	0.112				
A	129 (0.39)	180 (0.51)	1§	–				
G	201 (0.61)	172 (0.49)	1.18 (1.06–1.30)	0.002¶				

*11: homozygotes for the major allele, 12: heterozygotes and 22: homozygotes for the minor allele. ^†^ *n* = 165. ^‡^ *n* = 176. ^§^Reference category (odds ratio, 1.0). ^¶^Logistic regression analyses were used for calculating statistic value.

Haplotypes of NFE2L2 were constructed for the patients and controls, and six major haplotypes with frequencies >3% were identified (Table 5). The data showed no statistically significant difference in prevalence of haplotypes (H1, H2, H3, H4, H5, H6 and H7) between the T2DM patients and controls (*P* > 0.05).

**Table 5:** Frequency of haplotypes of NFE2L2 of T2DM patients and controls

Haplotypes	T2DM patients	Controls	*P*	OR (95% CI)
H1‐C A A C A A	17.95 (0.026)	18.16 (0.034)	0.47	0.78 (0.40–1.52)
e	273.60 (0.401)	231.04 (0.434)	e	0.91 (0.72–1.14)
H3‐G A A C G G	89.04 (0.131)	64.89 (0.122)	0.55	1.11 (0.79–1.57)
H4‐G A G C A A	152.64 (0.224)	112.61 (0.212)	0.47	1.11 (0.84–1.46)
H5‐G A G C G A	35.42 (0.052)	25.35 (0.048)	0.66	1.12 (0.67–1.90)
H6‐G G A C G A	21.65 (0.032)	15.03 (0.028)	0.67	1.16 (0.59–2.26)
H7‐G G A T G A	60.70 (0.089)	52.97 (0.100)	0.62	0.91 (0.62–1.34)

Order of polymorphisms: rs2364723, rs13001694, rs10497511, rs1806649, rs1962142, rs6726395. Global χ^2^ = 2.194, df = 6, *P* = 0.901. Haplotypes were omitted if the estimated haplotype probability was <3%.

### Serum HMOX1 levels and association with NFE2L2 SNPs

We randomly selected a subset of T2DM patients and control participants to evaluate serum HMOX1 levels with regard to its association with these NFE2L2 SNPs (Tables 6 and 7). The mean serum HMOX1 level of T2DM patients trends higher than that of the controls and was significantly high in patients with complications compared with those without complications (Table 6).

**Table 6:** Serum HMOX1 levels of the participants

	Participants, *n*	HMOX1 (ng/ml)	*P*‐value
Controls	53	0.29 ± 0.16	Reference
T2DM	111	0.36 ± 0.21	0.097
Without complications	84	0.35 ± 0.22	0.14
With complications	27	0.38 ± 0.17	0.048*

The Kruskal–Wallis test was used to analyse differences in serum HMOX1 levels, and then the *post hoc* Mann–Whitney *U*‐test. *P < 0.05, between T2DM patients with and without complications.

**Table 7:** Multivariate logistic regression analysis of association between clinical factors or NFE2L2 SNPs and serum HMOX1 levels in T2DM patients

	Estimate	Wald	*P*‐value	Point estimate OR (95% CI)
rs10497511 (AA *versus* AG+GG)	0.13	0.058	0.43	0.871 (0.242–2.668)
rs1962142 (GG *versus* GA+AA)	0.43	0.55	0.12	1.546 (0.963–4876)
rs6726395 (AG *versus* GG+AA)	−0.068	0.021	0.88	1.107 (0.426–2.689)
rs2364723 (GG *versus* CG+CC)	0.52	1.17	0.038	0.592 (0.230–0.152)
rs13001694 (AA *versus* AG+GG)	0.94	1.03	0.30	2.583 (0.417–16.001)
rs1806649 (CC *versus* CT+TT)	−0.67	0.41	0.52	0.534 (0.079–3.116)
Gender (male *versus* female)	0.52	1.27	0.25	1.681 (0.682–4.145)
T2DM complications (yes *versus* no)	0.27	0.33	0.86	1.315 (0.520–3.330)
Age (<60 *versus* ≥60)	−0.30	0.41	0.52	0.734 (0.287–1.881)

Multiple logistic regression analysis was performed to examine the correlations of HMOX1 level with NFE2L2 SNPs or other variables including gender, diabetic complication and age (Table 7). We found that only for rs2364723 there was a significant decrease in the serum HMOX1 levels in T2DM patients for the GG genotype carriers compared with participants with CG+CC genotype [0.592 (0.230–0.152), *P* = 0.038], suggesting that NFE2L2 polymorphism of rs2364723 may cause an increase in serum HMOX1 level in T2DM patients.

## Discussion

Nuclear factor erythroid 2‐like 2 is a transcription factor and functions to up‐regulate expression of antioxidant genes in response to oxidative stress 6, 13, 14, 15, 22, 23. We conducted this study to investigate NFE2L2 SNPs for possible associations with either T2DM or diabetic complications (including diabetic foot, nephropathy, retinopathy, microangiopathy and peripheral neuropathy) in a cohort of Chinese volunteers of Han descent. We did not find significant difference in genotype frequencies of these six SNPs between T2DM patients and healthy controls. However, T2DM patients with complications had a higher frequency of mutant rs2364723 C allele, rs10497511 G allele, rs1962142 A allele and rs6726395 G allele in the T2DM patients with complications than the T2DM patients without complications. Increased serum HMOX1 levels were also associated with mutant genotypes of rs2364723 in T2DM patients. Thus, this study suggests that NFE2L2 SNPs are associated with T2DM patients with complications and serum levels of HMOX1.

The first issue is that we did not find the significant difference between NFE2L2 SNPs and T2DM in this study. This may be related to the diversity of risks responsible for the development of T2DM, including diet, age, environmental alterations and genetic factors. Although these risks may exist with the presence of oxidative stress, the latter is not the pivotal step for the development of T2DM. In a published study, the association between different NFE2L2 SNPs and human cancers was established 24; however, there were also several studies that did not find the association between NFE2L2 polymorphisms and the risk of Alzheimer's 25, Parkinson's diseases 26 or oxidative stress biomarkers in patients with amyotrophic lateral sclerosis 27. Consistent with these previous studies, we found no association of NFE2L2 polymorphisms with the risk of T2DM. These results suggest that there was no significant association of NFE2L2 polymorphisms with these chronic diseases that were not predominantly caused by oxidative stress.

Although diabetic complications were developed based on diabetes, the susceptibilities of diabetic individuals to diabetes‐caused secondary complications are different. For instance, generally speaking the risk for heart disease is six times higher for women with diabetes, but only two‐ to threefold in men with diabetes, compared with those without diabetes 1, 2, 3, suggesting that except for diabetes there are other factors to also determine the risk of complication development in these diabetic individuals.

In addition, to our knowledge, T2DM can increase oxidative stress that is a major reason to induce T2DM complications 6, 13, 14; however, T2DM itself is caused by various factors among which oxidative stress may not be an essential one 1, 2, 3, 8. In a line with our finding, a few previous studies have shown that NFE2L2 polymorphisms were related to progression of Alzheimer's disease, although it was not associated with the risk of Alzheimer's disease 25. Similarly, although NFE2L2 polymorphisms were not associated with a susceptibility to childhood‐onset systemic lupus erythematosus, it could confer a risk in developing kidney malfunction in patients with the disease 28. These studies suggest that Alzheimer's diseases, childhood‐onset systemic lupus erythematosus and T2DM may be developed predominantly independent on the oxidative stress; however, their secondary effects on the organs such as various complications are related to the oxidative stress.

It has been well‐reported that the oxidative stress was increased in the patients with diabetes and diabetic animals, reflected by enhanced oxidative stress biomarkers in the bloods and tissues, such as malondialdehyde, 3‐nitrotyrosine, 4‐hydroxynonenal and reactive oxygen species 29, 30. The oxidative stress in the patients with T2DM may play a crucial role in the development of diabetic complications 23, 31. To support this concept, we demonstrated here that multiple NFE2L2 SNPs are associated with the development of various complications in T2DM patients caused by lack of the prevention of oxidative stress that has been appreciated as a key responsible factor for the development of diabetic complications 6, 8, 9, 12, 31.

At the cellular level, activation of NFE2L2 results in the up‐regulated expression of many cytoprotective proteins, including HMOX1. The latter is an essential enzyme in heme catabolism and has anti‐inflammatory properties *via* up‐regulation of IL‐10 and IL‐1R antagonist expression 32. It was reported that oxidative stress was increased and associated with the endothelial cell injury in HMOX1‐deficiency patients 33. Liu *et al*. 34 demonstrated that the absence of HMOX1 exacerbated myocardial ischaemia/reperfusion injury in diabetic mice, and expression of HMOX1 could ameliorate T1DM in mice 35. The relationship and associated pathways of NFE2L2 with downstream biomarkers have been reviewed 36. Normally, NFE2L2 locates in cytoplasm and binds to Keap1 remaining inactive. Under oxidative stress conditions, NFE2L2 is phosphorylated, released from Keap1 and transferred into the nuclei. Then the phosphorylated NFE2L2 binds to the ARE in the upstream promoter region of many anti‐oxidative genes such as HMOX1, and initiates their transcription 36. Thus, we speculate that serum HMOX1 levels might be negatively associated with the development of T2DM and/or its complications. Unexpectedly, however, our finding showed that the serum level of HMOX1 in the group of T2DM patients with complications was the highest (0.38 ± 0.17 ng/ml). In fact in patients with T2DM accompanied by kidney disease, the severity of renal failure was found to positively correlate with HMOX1 levels 37. In a line with this finding, a few other studies also demonstrated the association of the elevated plasma HMOX1 contents with the patients with impaired glucose regulation 38 and T2DM 39 in a Chinese population. In addition, HMOX1 protein levels in peripheral blood mononuclear cells were higher in the gestational diabetes than in the controls 40. Plasma levels of HMOX1were higher in patients with T2DM and tuberculosis and in patients with tuberculosis only 41. This positive association of serum HMOX1 with the several diseases was considered as the body's stress response with a potential to protect the oxidative stress induced by diseases such diabetes 37. This notion was supported by our experimental studies with animal models where we demonstrated the early increases and late decreases in cardiac expression of NFE2L2 and HMOX1, along with the late development of diabetic cardiomyopathy in the T1DM mouse model 42. The correlation analysis of serum HMOX1 and T2DM factors illuminated that only mutant rs2364723 G carriers could significantly decrease the serum HMOX1 levels in T2DM patients, which supports the hypothesis we described above.

Limitations of this study include the high number of non‐informative samples, which was because of inadequate samples or PCR readings. Moreover, our study population was limited to the geographic area of Northeast China, which contains certain specific factors such as temperature (cold at winter and cool at summer compared with Southern populations of China), dietary and environmental differences, and even genetic background. All these may cause certain impacts on the serum antioxidant or oxidative stress marker patterns. In addition, we also found a significantly higher plasma level of HMOX1 in those carrying with the rs2364723 CG or CC allele than in those carrying the rs2364723 GG allele. As a result of relatively small sample size specifically for this observation in this study, we could not have exact explanation now. Thus, further studies with a larger sample size including diverse populations from different geographic areas are needed to verify differences relative to controls.

In summary, this study investigated the association of NFE2L2 polymorphisms with T2DM and its complications. We found that multiple mutations of NFE2L2 rs2364723, though not associated with T2DM, were significantly associated with the prevalence of complications in T2DM, indicating that this gene locus may predispose towards diabetic complications. The mutation of NFE2L2 rs2364723 G allele was significantly associated with increased serum HMOX1 levels in T2DM patients.

## Conflict of interest

The authors have no conflicts of interest to declare for this work.

## Author contribution

Lu Cai and Lining Miao initiated and designed the study. Xiaohong Xu, Jing Sun, Xiaomin Chang, Ji Wang and Manyu Luo participated in recruiting samples and/or performed laboratory studies. Lu Cai, Lining Miao and Kupper A. Wintergerst periodically discussed the progression of project, data interpretation and wrote the draft of manuscript. Lu Cai and Kupper A. Wintergst revised and formed the final manuscript. All authors contributed to review of the draft and revised manuscript with certain suggestions.
